# The Alpine Cushion Plant *Silene acaulis* as Foundation Species: A Bug’s-Eye View to Facilitation and Microclimate

**DOI:** 10.1371/journal.pone.0037223

**Published:** 2012-05-24

**Authors:** Olivia Molenda, Anya Reid, Christopher J. Lortie

**Affiliations:** 1 Ecology and Evolutionary Biology, University of Toronto, Toronto, Ontario, Canada; 2 Department of Biology, York University, Toronto, Ontario, Canada; WSL Institute for Snow and Avalanche Research SLF, Switzerland

## Abstract

Alpine ecosystems are important globally with high levels of endemic and rare species. Given that they will be highly impacted by climate change, understanding biotic factors that maintain diversity is critical. *Silene acaulis* is a common alpine nurse plant shown to positively influence the diversity and abundance of organisms–predominantly other plant species. The hypothesis that cushion or nurse plants in general are important to multiple trophic levels has been proposed but rarely tested. Alpine arthropod diversity is also largely understudied worldwide, and the plant-arthropod interactions reported are mostly negative, that is,. herbivory. Plant and arthropod diversity and abundance were sampled on *S. acaulis* and at paired adjacent microsites with other non-cushion forming vegetation present on Whistler Mountain, B.C., Canada to examine the relative trophic effects of cushion plants. Plant species richness and abundance but not Simpson’s diversity index was higher on cushion microsites relative to other vegetation. Arthropod richness, abundance, and diversity were all higher on cushion microsites relative to other vegetated sites. On a microclimatic scale, *S. acaulis* ameliorated stressful conditions for plants and invertebrates living inside it, but the highest levels of arthropod diversity were observed on cushions with tall plant growth. Hence, alpine cushion plants can be foundation species not only for other plant species but other trophic levels, and these impacts are expressed through both direct and indirect effects associated with altered environmental conditions and localized productivity. Whilst this case study tests a limited subset of the membership of alpine animal communities, it clearly demonstrates that cushion-forming plant species are an important consideration in understanding resilience to global changes for many organisms in addition to other plants.

## Introduction

Facilitation, or positive interactions between organisms that benefit at least one species and are harmful to neither, is relatively common in most plant communities [1], [2] and frequent in stressful climates [3], [4]. Processes such as facilitation integral to community assembly are important to consider in light of current ecological issues such as global change, biodiversity, and ecosystem sustainability because biotic interactions may change their impacts. In order to better understand community assembly, a critical assessment of the scope of facilitation is thus needed - particularly in harsh environments [4]. Positive interactions in general have significant impacts on community organization, dynamics, and productivity [5], but the major advances to date in the facilitation literature have been primarily focused on plant-plant interactions and within a given trophic level [4], [6]. Plant-invertebrate facilitation studies are extremely rare; existing studies can be categorized as plant-pollinator or plant-ant facilitation and both categories are well established in a variety of climates [7], [8], [9], [10], [11], [12], [13]. For instance, in the journal ‘Arthropod-Plant Interactions’, 65% of all articles published described negative impacts of arthropods on plants such as parasitism and herbivory, 24% focused on pollinators, 10% dealt with global concerns such as diversity, climate change, and technological advances, whilst only 1% of all articles published described positive interactions between arthropods and plants (inspection of all abstracts published in this journal up to 2011). A significant component of ecological interactions are thus being overlooked. Communities in an ecological-change context are comprised of plants, microbes, and invertebrates. It is thus critical for the field of facilitation to incorporate other trophic levels. To do so, it must encompass interactions at some of these additional trophic levels and explore whether facilitation is relevant to community assembly and arthropod-plant interactions. We propose that the logical first step in developing novel implications to these theories is to identify and document the positive interactions between taxa including more than one trophic level.

In the alpine, facilitation frequently occurs in the form of nurse plants that modify the environment by reducing physical stress or disturbance thereby allowing less tolerant plant species to survive [4], [14], [15], [16], [17], [18]. Nurse plants frequently increase plant species richness [19] but not always [20], and cushion plants are likely the dominant form for nurse plant species in the alpine [21]. The structure of their canopy is genetically determined and grows as a dense dome that traps heat, moisture, and nutrients providing them with the ability to moderate harsh alpine conditions because it minimizes the negative effects of wind and low temperatures [22]. As a result, cushion plants are commonly classified as ecosystem engineers in the alpine [5], [19], [21], [23]. With increasing habitat loss due to climate change, cushion plants can thus be a critical first step in assessing the responsiveness of a community to change. Cushions consistently increase species richness at the entire plant community level and can similarly increase biomass [24], [25]. Less frequently tested, cushion plants can also positively influence other taxa such as ladybird abundance in the Andes [26], [27]. As alpine surfaces are released from glaciations, arthropod predators invade and depend on invertebrates that arrive with upward winds [28]. Other invertebrates can only colonize once a plant system is established; as a result, alpine invertebrate communities are determined by the structure of local plant communities [10], and since cushions are fundamental to enhancing plant diversity, it is reasonable to assume that these effects scale up to other trophic levels. Cushion plants are thus the ideal set of species to explore the relative importance of positive plant-arthropod interactions on the assembly of alpine communities.

Here, we test the overarching hypothesis that the nurse plant effect of alpine cushions on other plant species extends to the entire invertebrate community – not just target species such as ants or bees. Hence, this case study examines the capacity for cushions to serve as the more broadly defined foundation species which are species at lower trophic levels, locally and regionally abundant, and fundamental to some aspects of ecosystem function such as diversity maintenance [29], [30], [31]. Importantly, cushion plant loss in the alpine with a changing climate would dramatically impact ecosystem stability and species diversity for many taxa [30]. To examine this hypothesis, we test the following predictions using one of the most common alpine cushion species, *Silene acaulis*: (1) similar to previous studies, that this cushion is a nurse for other plants in our system, (2) that this cushion increases the abundance and diversity of invertebrates relative to microsites with other vegetation, and (3), more generally, that microhabitat is a critical factor influencing the overall structure of plant-invertebrate distribution in the alpine.

## Methods

### Species and Site Descriptions

**Figure 1:**
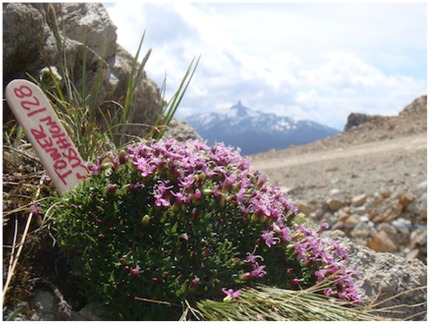
Female *Silene acaulis* in bloom on Whistler Mountain. Photo taken 7-21-2010, field of view, 25×15 cm.

The study species *Silene acaulis* is a cushion-forming gynodioecious plant [32], and the most widespread alpine cushion plant in the Northern Hemisphere [22]. This species is frequently present in the Fitzsimmons Mountain Range in British Columbia, Canada (GBIF search, http://data.gbif.org). It generally grows on wind-exposed ridges, rocky slopes, and open alpine grasslands between 1700 and 2400 m in elevation [33]. *S. acaulis* can survive extreme temperatures from −80 to 60°C [33], and the dense, dome shaped structure (Figure 1) has been shown to moderate temperature, reduce wind, increase moisture, and increase soil nutrients [22], [34].

Four *S. acaulis* sites were sampled on Whistler Mt. in British Columbia, Canada, and site attributes were recorded including lat and long (+/−10 m), elevation, slope, and relative cover of dominant substrate classes (Table 1). The average alpine temperature as recorded by the Whistler-Blackcomb resort is −8 to 5°C over the summer (listed on website as a long-term mean: www.whistlerblackcomb.com). Sites were delineated by a minimum density of 80 individuals of *S. acaulis* within a defined and permanently marked 100×100 m area. No specific permits were required for the described field studies. No specific permissions were required for these locations/activities since the location is not privately owned nor protected in any way. The field studies also did not involve endangered or protected species.

**Table 1:** A description of the study sites selected on Whistler Mountain used to test for the effects by *Silene acaulis* on the plant and invertebrate communities.

Site	GPS	Elevation (m)	Slope (%)	Habitat
1.	50 ? 03′35.11″N 122 ? 57′33.21″ W	2164	50	73% Rock 22% vascular 4% lichen 1% cushion
2.	50 ? 03′ 31.27″N 122 ? 57′21.81″ W	2165	72	72% Rock 18% vascular 8% lichen 1% cushion
3.	50° 03′470′′ N 122° 57′ 506′′ W	2152	23	48% rock 45% vascular 6% lichen 1% cushion
4.	50 ? 03′32.53″N 122 ? 57′30.43″W	2168	61	instrumentation

Vegetation and arthropod sampling were conducted at sites 1–3 whilst the 4^th^ site was used for microclimatic instrumentation.

### Experimental Design

Parallel linear transects (placed at random distances apart) were used to select cushions within the sampling area defined at each site. Every cushion that intersected the transect was used for a total of 135 cushions (approximately 35 at each site). A paired adjacent non-cushion or ‘open’ microsite was selected not more than 0.5 m to the right (facing North) of each marked cushion plant carefully controlling for the same slope and aspect. Microsites were defined as elliptical plots (or microsites as termed herein), and the longest axis and perpendicular axis measured on the cushion and the equivalent dimensions and area were sampled at the paired, vegetated locations. Plant species richness, density, surface area, number of flowers, and aspect were recorded at each microsite – both cushion and open. Only plants rooted in *S. acaulis* were counted which likely underestimates its relative effect. Arthropod diversity was collected by vacuuming each microsite (cushion or paired microsite with other non-cushion vegetation) using the Dirt Devil Gator © 18 V handheld vacuum for 1 minute. This effectively sampled insects from the surface of plants, within the vegetation, and under the leaves but not belowground. The insects were then deposited into ziplock bags and frozen for subsequent processing. All arthropods were sampled during sunny, warm weather above 5°C between 11∶00 - 13∶00 hrs.

On August 15^th^, 2010, all 270 microsites were sampled for estimates of the boundary layer by recording the average height of the vegetation. Specifically, the maximum and minimum height was measured in addition to 3 haphazardly selected height measurements per plant. In order to quantify the type of habitat surrounding each microsite, a 0.5×0.5 m grid of ten squares was created. The grid was centered and placed over the entire elliptical plot of the microsite. The substrate type that dominated each square was classified using the following classes: rock, vascular vegetation, non-vascular vegetation, or cushion plant. Microclimate was also recorded to assess the relative capacity of cushions to modify local conditions. So as to avoid interference with insects, 12 additional cushion and open pairs were selected for instrumentation. A HOBO ProV2 external temperature and relative humidity data logger was placed in each microsite. In cushion microsites, the sensors were placed directly inside the cushion. In the paired open vegetation sites, the sensors were placed under vegetation or litter. Temperature and relative humidity was logged at 30-minute intervals from 7/17/2010 to 8/25/2010.

### Statistical Analyses

Arthropod and plant diversity indices were calculated for each microsite using EstimateS 8.2.0 including rarefaction curves of the Mao Tau estimates for mean species richness per sampling class [35]. Conventional metrics were used to study community diversity and composition including species richness, Simpson’s index, and abundance [36], [37]. Functional richness of plants and arthropods was also examined. Plants were classified based on life-form including grasses, herbs and forbs, sedges, shrubs, and lichens [38]. Arthropods were classified by order which in the alpine closely parallels diet or feeding mode, i.e. spiders, flies, mites and ticks, grasshoppers, beetles, etc. [9].

A Pearson’s chi-squared test for *r* x *k* contingency tables was used to test if the distribution of insect and plant species differed between microsites, and residuals were examined to determine important associations and direction [39]. A detrended correspondence analysis (DCA) was used to compare community composition between microsites and in response to the environmental gradients measured, and statistically significant differences were identified with multiple response permutation procedures [40]. To prevent leverage effects of rare species, rare species sampled in less than three microsites were excluded [39]. Generalized linear mixed effects models (GLMMs) were used to test for effects of treatment (cushion or open microsites), site, surface area, boundary layer height, or aspect on plant and arthropod abundance and diversity measures. Site was coded as a random effect. Both arthropod and plant data were examined for overdispersion [41]. Neither was overdispersed, and therefore a Poisson distribution was selected [41]. Tukey HSD post hoc contrasts (multiple, linear) were used where appropriate to identify differences between specific categorical factor levels (alpha at p<0.05) and post hoc regressions for continuous factors. To enable contrasts between plant responses to cushions and arthropods and to assess the biological importance of statistically significant differences, the relative intensity of interactions (Rii) effect size metric was also calculated with cushion designated as the treatment and open as the control and compared via t-tests [42]. This metric is symmetric around 0, ranges from −1 to +1, and negative values denote relative competition whilst positives denote facilitation. The GLMMs were conducted using the lme4 package in R.2.10.1, and all other statistical analyses were conducted using R. 2.10.0.

The 12 pairs of samples (cushion – open microsites) were averaged for each half hour period from 7/17/2010 to 8/25/2010 to produce a microclimatic profile for the growing season (n = 1859 instances). While this approach reduces data, it reduces the likelihood of Type I error due to pseudoreplication [43]. Descriptive statistics were calculated for both microclimatic data sets, and a normal climate range was established for each microsite type by taking two standard deviations above and below the mean since this corresponds to 95% of the area under a curve [39]. Both data sets were plotted on the same graph, and the climate data for non-cushion microsites was analyzed for deviations above or below the normal climate range for the cushion microsites. Deviations that exceeded this range were considered statistically significant.

## Results

### Plant and Arthropod Community Patterns

**Figure 2:**
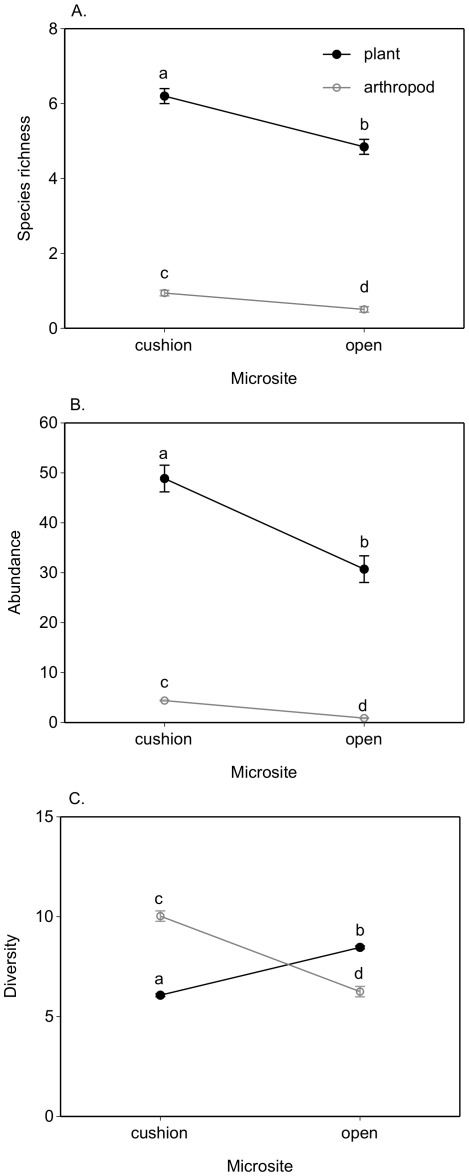
The mean community composition estimates for plants and arthropods associated with cushion plants and open non-cushion vegetated microsites. The mean +/−1 s.e. are denoted (significant Tukey post hoc contrasts denoted by different letters). Please see text for full details of response variables.

**Figure 3:**
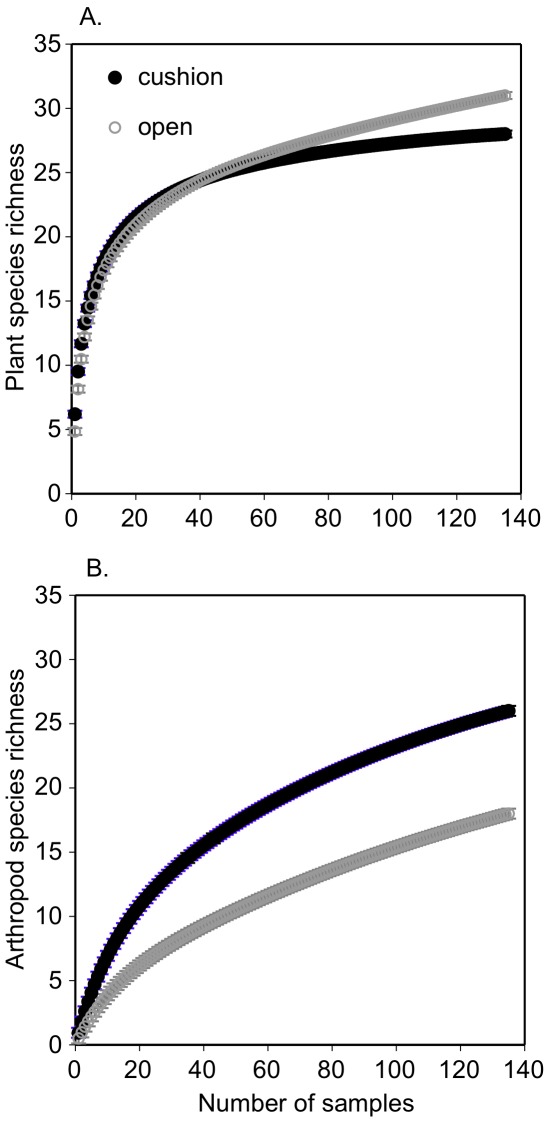
Rarefaction curves for plant and arthropod species sampled on alpine cushion plants and paired open non-cushion plant vegetation. Mao Tau estimator and +/−1 s.e. are shown.

**Figure 4:**
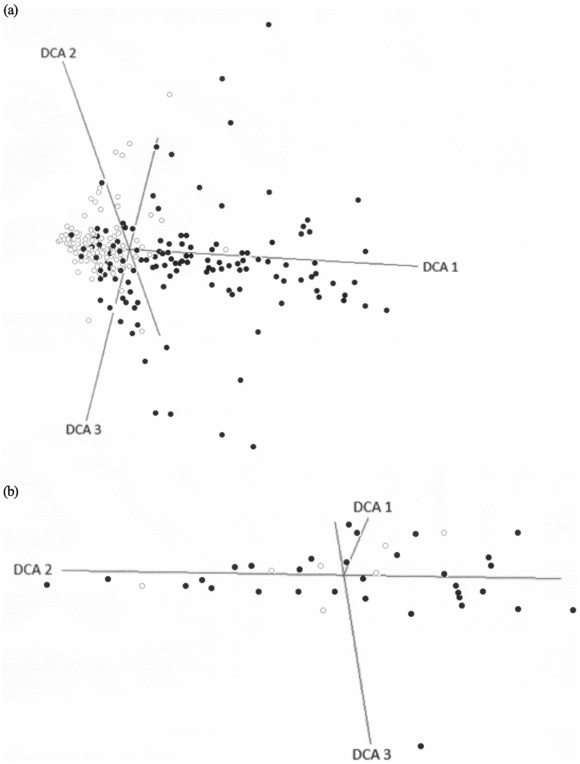
Three space ordination plots from detrended correspondence analyses for plant and arthropod richness sampled at three alpine sites on cushions and paired open non-vegetated microsites. Black points represent cushion microsites while open grey points show open microsites.

There were significantly more plants and plants species found on cushion plants relative to the paired non-cushion microsites (Figure 2AB, Table 2). However, Simpson’s diversity index was significantly greater at open or non-cushion microsites (Figure 2C, Table 2). Plant species also accumulated more quickly in the paired open microsites (Figure 3A), but 68% of plant species were more frequently sampled on cushions (Chi-square = 237, df = 32 p = 0.0001, Figure S1). There were significantly more arthropods, more arthropod species, and higher diversity associated with cushions (Figure 2, Table 3). Arthropod species accumulated more rapidly on cushions relative to open microsites (Figure 3B), and 72% species were sampled more frequently on cushions (Chi-square = 122, df = 33, p = 0.0001, Figure S2; Table S1 lists all abbreviations). The functional richness of plants and arthropods was significantly greater on cushions relative to open microsites (GLMM, Chi-square_plant functional richness_ = 9.8, df = 1, p = 0.001, Chi-square_arthropod functional richness_ = 16.2, df = 1, p = 0.0001; no other factors were significant in these models); however, lichens preferred open microsites while Araneida (spiders) were found in both microhabitats. The plant community composition was different at each of the three sites and between microsites (MRPPs p_site_ = 0.001, p_microsites_ = 0.001, Figure 4A) whilst arthropod community assemblages varied similarly (MRPPs p_site_ = 0.004, p_microsites_ = 0.03, Figure 4B). The relative importance of cushions to arthropods versus to other plants was significantly greater (Tables 2 & 3, Rii columns list mean effect sizes, post hoc t-tests for differences, all p<0.05). The positive effect of cushions on arthropod richness doubled relative to plants, abundance effects were 40% greater, and diversity effects were 66% greater on average.

**Table 2:** A summary of the GLMMs used to test the importance of treatment (cushion-open) and physical factors on estimates of alpine plant community composition.

Measure	Factor	DF	Chi-square	P> Chi-square	Post hoc	Rii
Richness	Treatment	1	22.7	**0.0001**	C > Op	0.12+/−0.02
	Site	2	1.5	0.5		
	Treatment x Site	2	2.1	0.35		
	Surface area	1	10.1	**0.002**	+	
	Boundary layer	1	11.7	**0.0006**	+	
	Aspect	7	14.8	**0.04**	S > W	
Abundance	Treatment	1	561	**0.0001**	C > Op	0.23+/−0.03
	Site	2	17	**0.0002**	1>2,3	
	Treatment x Site	2	3.1	0.21		
	Surface area	1	651	**0.0001**	+	
	Boundary layer	1	213.8	**0.0001**	+	
	Aspect	7	203	**0.0001**	S > W	
Diversity	Treatment	1	53.3	**0.0001**	C < Op	−0.15+/−0.009
	Site	2	1.1	0.6		
	Treatment x Site	2	0.31	0.85		
	Surface area	1	0.007	0.93		
	Boundary layer	1	0.43	0.51		
	Aspect	7	0.63	0.99		

Richness is the number of species, abundance the total number of individuals, and diversity the Simpson’s index. Tukey post hoc contrasts were used to assess categorical, within factor level differences (i.e. C  =  cushion or Op  =  open) and regressions used for continuous (+ indicates significant positive relationship). The degrees of freedom listed refer to the specific factor (DF_model_ = 14). Bold denotes significance at p<0.05. The mean Rii is reported +/−1 s.e. to show strength of effect.

**Table 3:** A summary of the GLMMs used to test the importance of treatment (cushion-open) and physical factors on estimates of alpine arthropod community composition.

Measure	Factor	DF	Chi-square	P> Chi-square	Post hoc	Rii
Richness	Treatment	1	18.5	**0.0001**	C > Op	0.36+/−0.07
	Site	2	3.7	0.16		
	Treatment x Site	2	0.92	0.63		
	Surface area	1	0.14	0.71		
	Boundary layer	1	3.1	0.08		
	Aspect	7	4.2	0.76		
Abundance	Treatment	1	21.8	**0.0001**	C > Op	0.32+/−0.05
	Site	2	37.6	**0.0001**	1>2,3	
	Treatment x Site	2	8.7	**0.013**		
	Surface area	1	0.05	0.84		
	Boundary layer	1	0.008	0.93		
	Aspect	7	3.3	0.85		
Diversity	Treatment	1	122.2	**0.0001**	C > Op	0.25+/−0.01
	Site	2	18.9	**0.0001**	2>1	
	Treatment x Site	2	3.7	0.16		
	Surface area	1	0.007	0.93		
	Boundary layer	1	2.7	0.1		
	Aspect	7	13.2	0.07		

Richness is the number of species, abundance the total number of individuals, and diversity the Simpson’s index. Tukey post hoc contrasts were used to assess categorical, within factor level differences (i.e. C  =  cushion or Op  =  open). The degrees of freedom listed refer to the specific factor (DF_model_ = 14). Bold denotes significance at p<0.05. The mean Rii is reported +/−1 s.e. to show strength of effect.

### Mechanisms and Microclimate

Plant species richness and abundance were effectively described by surface area sampled, boundary layer height, and aspect while Simpson’s diversity was not related to these physical factors (Table 2). Surface area sampled and boundary layer positively predicted plant richness and abundance (Table 2 with post hoc regressions). The DCA also suggested that there were multiple factors driving plant species composition (Table 4, significant factor correlations listed). Arthropod richness and abundance were however significantly described the by biotic factor plant abundance (GLMM, Chi-square_arthropod richness_ = 24, df = 1, p = 0.0001, Chi-square_arthropod abundance_ = 5.7, df = 1, p = 0.017). Nonetheless, there were still significant gradients shaping the entire assemblage of arthropods including boundary layer and aspect – south (Table 4).

**Table 4:** Detrended correspondence analyses for plant and arthropod richness patterns.

	Plant species				Arthropod species			
	Axis 1	Axis 2	Axis 3	Axis 4	Axis 1	Axis 2	Axis 3	Axis 4
Eigenvalues	0.4262	0.332	0.3491	0.2853	1	1	0.8680	0.7630
Axis lengths	3.5409	3.6933	3.8056	4.3192	1	1	6.9665	3.8842
Correlation with Factors								
Cushion Microsites	**0.3092**	**−0.1213**			**0.0089**	**−0.2424**		
Open Microsites	**−0.1547**	**0.0607**			**−0.0161**	**0.4414**		
Sampling Date	**−0.95050**	**−0.31074**			**0.9953**	**−0.09587**		
Site (2)	**−0.0767**	**0.0262**			n/a	n/a		
Surface Area	**0.96461**	**0.26369**			n/a	n/a		
Boundary Layer	**0.99028**	**0.13909**			**−0.9814**	**−0.19153**		
Aspect – South	n/a	n/a			**−0.9814**	**−0.19153**		

Bold denotes significant correlations between factors and ordination axes at p≤0.05, and n/a indicates no relationship. See text for description of factors.

**Figure 5:**
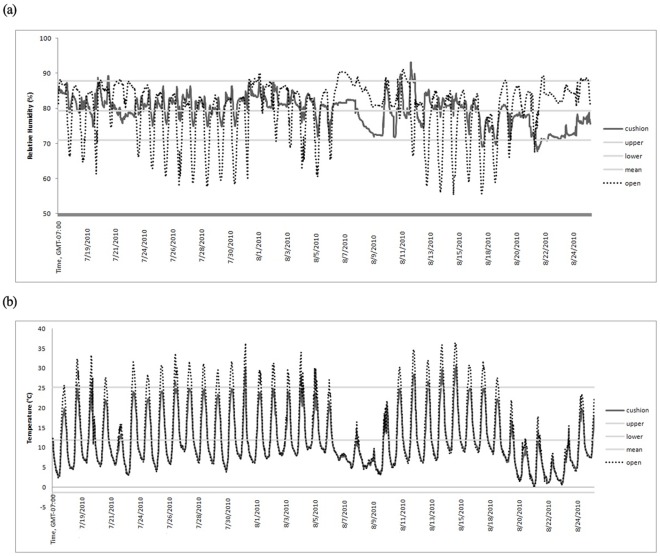
Microclimatic profiles on alpine cushion plants and paired open non-cushion vegetation microsites (n = 12) throughout the 2010 growing season. The mean relative humidity (%) and temperatures (°C) for cushions are shown by the light grey lines (mean ±2 σ). Rain events are shown as the amount of precipitation (mm).

*S. acaulis* cushions maintained a stable relative humidity over the summer which was similar to open microsites but far less variable (mean_cushion_ µ = 79.4%, σ = 4.2%, mean_open_ µ = 79.8%, σ = 8.1%, Figure 5A). During precipitation-free periods, humidity dropped during maximum solar gains causing a distinct diurnal pattern, whilst during precipitation events, relative humidity increased more in open microsites than in cushions (Figure 5A). A total of 25.5% of recorded instances in the open were significantly different from the cushion normal humidity levels (p≤0.05) with the majority of this difference driven by lower humidity. Temperature in cushions averaged 11.9±6.6°C over the summer whilst temperatures in open microsites averaged 13.2±8.5°C. Temperature also cycled in a diurnal pattern (Figure 5B). Open areas experienced higher daily maximum temperatures than cushions but similar minimum temperatures (Figure 5B). From all observations made in open microsites throughout the summer, 14.3% were significantly higher than cushion normal summer temperatures (p≤0.05).

## Discussion

Facilitation can dramatically reshape community theory [1], [44], and typical plant facilitation studies must now include a wider range of organisms or ‘scale up’ in some other respect to fully assess the import of these interactions for synthesis. Hence, we hypothesized that plant facilitation can extend to the larger community to include other trophic levels such as invertebrates, and whilst this may seem obvious, it is largely untested. We confirmed that *S. acaulis* does facilitate other plant species and more importantly that it also enhances all community measures tested for the alpine invertebrates. The composition of both plant and arthropod species was unique, diverse, and consisted of rare species (actually most species) which also supports the larger scope of interpretation we developed in brief herein that nurse plants can sometimes serve as foundation species in relatively simple systems such as the alpine. Hence, both direct and indirect effects can cascade up from these species to enhance both the diversity and complexity of the interaction webs in the alpine due to cushion plants. As indicated, we are certain that this is a strong first step in speaking to theories predicated upon these interactions such as realized niches [45], [46], extended phenotypes [47], or climate-envelope theories associated with change [48] to name a few. The final prediction tested that microhabitat in general is a critical factor to consider in describing alpine plant-invertebrate distributions was supported which suggests that cushion plants do filter larger climatic factors and that unfortunately a changing climate may shift these complex interactions in ways very difficult to predict. The next step is to link interaction studies such as this one to function and assembly in the alpine given that nurse plants can likely be promoted to a foundational status in these ecosystems.

The positive effect by *S. acaulis* on the plant community has been observed for this species in other ranges which suggests that these findings are not context dependent nor locally restricted but apply to regional or even ecosystem-level assembly processes in many alpine ecosystems. Cavieres and Badano (2009) and Antonsson et al. (2009) similarly observed *S. acaulis* effects on vascular plant richness in the Andes and Northern Sweden whilst Quiroz et al. (2009) found the identical effects to those we detected in BC, Canada namely that vascular plant richness and abundance were higher on cushions but that other measures of diversity such as evenness or the Simpson’s index were higher in the open. This is an intriguing finding which suggests that whilst the positive effect of this cushion is important and consistent not all species uniformly capitalize on the niche it provides – i.e. open sites are less favourable in many respects but they do provide a wider range of available microclimatic types. Hence, cushions are a key player in maintaining the diversity of other plant species in the alpine by buffering fluctuations, but this conclusion must be tempered by the fact that more open sites with other vegetation provide viable and more broadly diverse sets of environmental conditions. Importantly, this suggests that detailed studies of dispersal and demography on and off cushions would further elucidate whether they are sources or sinks for various other plant species [48]. The diversity of plants we observed growing on *S. acaulis* was also similar to the assemblies observed in climate warming studies [49], [50], [51], and given that vascular plants are assumed to have a competitive advantage over non-vascular plants in milder climates, competition between plants growing on cushions may exclude some species including non-vasculars from these microsites (as was the case here). Climate change experiments have demonstrated that tundra communities when released from harsh conditions can sometimes become dominated by deciduous vascular vegetation excluding evergreen and non-vascular plants [49]. Alpine vegetation often grows in clumps to mitigate harsh climate [22], but on *S. acaulis* cushions, species were often capable of growing as single individuals in this system. Consequently, these cushions have more rare species present per unit area than comparable open areas. Cushions therefore have a profound impact on plant community organization and productivity by increasing diversity and abundance but a fuller understanding of the extent of their effects on plant community assembly could include demography, dispersal, and competition studies.

The facilitative signal by this cushion plant was even more dramatic for the invertebrate community with higher richness, abundance, and diversity and much larger effect size estimates. This can be explained by the fact that invertebrate communities are structured into two distinct groups - predators which live free of habitat requirements and all other insects which are determined by the plant community present [10], [28], [52], [53], [54]. Hence, there was an abundant yet low diversity group of predators (Arachnids) living in the open, and a highly diverse and abundant group of all orders of invertebrates living on cushions in this alpine system. Given the harsh conditions, specific alpine plant species can thus host unique invertebrate communities, and at the very minimum, *S. acaulis* can be considered an umbrella species whose presence facilitates community-wide diversity [55]. Plant species richness was a dominant driver of the cushion effect on arthropods detected here. This suggests that, in part, the effect of cushion plants is mediated through an indirect effect of facilitating other plant species that in turn provides a diverse palette of resources for the invertebrate community. The synergy of this facilitation also explains the much larger effect sizes associated with cushion effects on arthropods versus plants since the direct and indirect positive effects are summed for the arthropods and not for the plants. Plant species richness generally increases arthropod diversity and strongly shapes trophic structure [56]. Hence, cushion plants likely not only provide refuges and resources but also provide complexity upon which food and interaction webs can occur. Foundation species in stressful environments often have these more comprehensive effects [57], [58] so it is reasonable to assume that the plant-arthropod pattern we observed mirrors potential trophic complexity. This suggests that cushions may not only be nurse plant species but an umbrella for conservation and a foundation for ecosystem function in the alpine. Diet to assess food webs, demography of arthropods on and off cushions, and invertebrate dispersal patterns would be highly novel platforms of research given these findings.

The final prediction explored in this study was that fine-scale microclimate provides a critical set of factors to consider in explaining plant-arthoprod distribution in the alpine. Both plant and invertebrate diversity were influenced by microclimate. Aspect, south facing (within a given meadow based on microtopography) enhanced arthropod richness and boundary layer of the vegetation enhanced both plants and arthropods. Southern aspects are generally warmer, and a relatively larger boundary layer further reduces climate stressors [59], [60], [61]. Given that invertebrate diversity increased with plant diversity, decreasing rock and other non-vegetation cover classes at the microhabitat-level are important. Hence, vegetation in general has a positive effect on arthropods in the alpine. Within *S. acaulis*, the effects are of course more dramatic. The cushions mediated daily high temperature peaks and relative drops in humidity for the plants and invertebrates living on and inside it. Alpine cushions in general can act as heat traps maintaining temperatures up to 15°K warmer than ambient air while simultaneously serving as moisture traps rarely experiencing less than −0.6 MPa [22]. Other studies have shown that *Azorella compacta*, a tropical alpine cushion plant, has reduced oscillations in internal temperature relative to surface temperature and greater water potentials than alpine mat forming species [62]. Badano et al. (2010) also found that *Azorella monantha* (a similar cushion forming species) buffered extreme temperatures and increased soil moisture. However, we found that *S. acaulis* buffers only high temperatures and low humidity - acting more as an air conditioning system than a heat trap in our rocky alpine systems. The sensors in this study were also placed inside open vegetation and not simply on the soil surface but this likely only underestimates the relative differences between cushions and open microsites. Plants and invertebrates living on *S. acaulis* thus likely suffered less heat stress and certainly less drought stress than in the open. By promoting higher plant productivity, cushions generate a larger boundary layer that amplifies their importance as micro-scale filters of climate. Hence, a plant and bug’s-eye view using cushions in the alpine is an excellent launching point for understanding climate change effects on community assembly.

## Supporting Information

Figure S1 **The frequency of occurrence of species of plant species on cushion plants and paired open, vegetated sites.**
(TIF)

Figure S2 **The frequency of occurrence of species of arthropod species on cushion plants and paired open, vegetated sites.**
(TIF)

Table S1 The list of abbreviations associated with frequency of occurrence plots.
(DOC)
