# Supplementary figures and images for: The Alpine Cushion Plant Silene acaulis as Foundation Species: A Bug’s-Eye View to Facilitation and Microclimate
Source: PLoS One. 2012 May 24;7(5):e37223. doi: 10.1371/journal.pone.0037223 (PMC3360034; doi:10.1371/journal.pone.0037223)

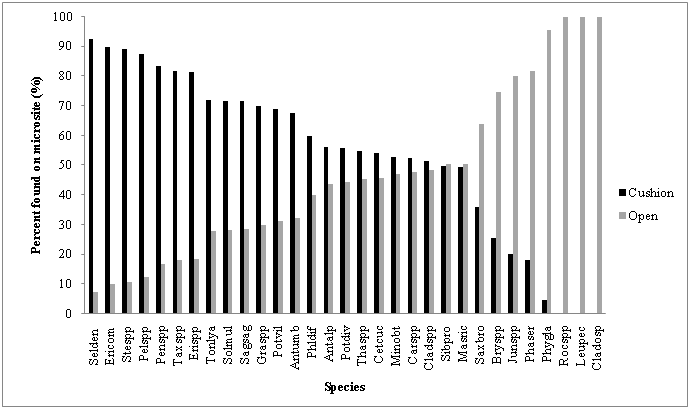

Supplement: Figure S1 — The frequency of occurrence of species of plant species on cushion plants and paired open, vegetated sites. (TIF) [file pone.0037223.s001.tif]

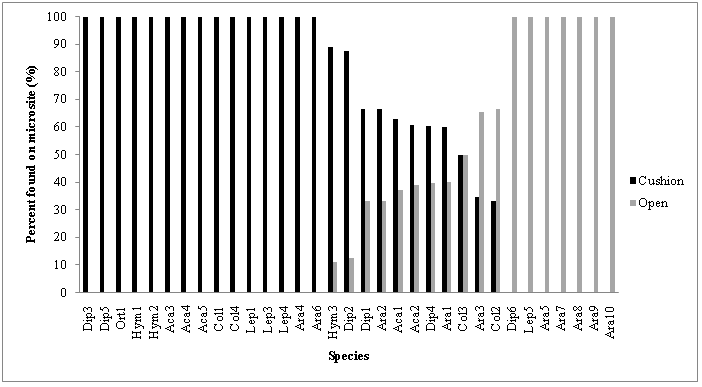

Supplement: Figure S2 — The frequency of occurrence of species of arthropod species on cushion plants and paired open, vegetated sites. (TIF) [file pone.0037223.s002.tif]
