# Supplementary material for: The Alpine Cushion Plant Silene acaulis as Foundation Species: A Bug’s-Eye View to Facilitation and Microclimate
Source: PLoS One. 2012 May 24;7(5):e37223. doi: 10.1371/journal.pone.0037223 (PMC3360034; doi:10.1371/journal.pone.0037223)
Supplement: Table S1 — The list of abbreviations associated with frequency of occurrence plots. (DOC) [file pone.0037223.s003.doc]

**Table S1.** The list of abbreviations associated with frequency of occurrence plots.

| **Plant abbreviation** | **Order** | **Genus** | **Species** | **Arthropod abbreviation** | **Order** |
| --- | --- | --- | --- | --- | --- |
| Ast1 | Asterales | Antennaria | alpina | Aca1 | Acarina |
| Ast2 | Asterales | Antennaria | umbrinella | Aca2 | Acarina |
| Ast3 | Asterales | Erigeron | compositus | Aca3 | Acarina |
| Ast4 | Asterales | Erigeron | sp. | Aca4 | Acarina |
| Ast5 | Asterales | Solidago | multiradiata | Aca5 | Acarina |
| Ast6 | Asterales | Taraxacum | sp. | Ara1 | Araneida |
| Ast7 | Asterales | Tonestus | lyallii | Ara2 | Araneida |
| Bra1 | Brassicales | Draba | lochocarpa | Ara3 | Araneida |
| Bry1 | Bryophyta | Moss | sp. | Ara4 | Araneida |
| Car1 | Caryophyllales | Minuartia | obtusiloba | Ara5 | Araneida |
| Car2 | Caryophyllales | Sagina | saginoides | Ara6 | Araneida |
| Eri1 | Ericales | Phlox | diffusa | Ara7 | Araneida |
| Eri2 | Ericales | Phyllodoce | glanduliflora | Ara8 | Araneida |
| Eua | Euasterid | Phacelia | sericea | Ara9 | Araneida |
| Lam1 | Lamiales | Penstemon | sp. | Ara10 | Araneida |
| Lec1 | Lecanorales | Cetraria | cucullata | Col1 | Coleoptera |
| Lec2 | Lecanorales | Cladina | sp. | Col2 | Coleoptera |
| Lec3 | Lecanorales | Cladonia | sp. | Col3 | Coleoptera |
| Lec4 | Lecanorales | Masonhalea | richardsonii | Col4 | Coleoptera |
| Lec5 | Lecanorales | Stereocaulon | sp. | Col5 | Coleoptera |
| Lec6 | Lecanoromycetes | Crustose | sp. | Dip1 | Diptera |
| Pel1 | Peltigerales | Peltigera | sp. | Dip2 | Diptera |
| Per1 | Pertusariales | Thamnolia | sp. | Dip3 | Diptera |
| Pin1 | Pinales | Juniperus | sp. | Dip4 | Diptera |
| Poa1 | Poales | Carex | sp. | Dip5 | Diptera |
| Poa2 | Poales | Grass | sp. | Dip6 | Diptera |
| Ros1 | Rosales | Luetkea | pectinata | Dip7 | Diptera |
| Ros2 | Rosales | Potentilla | diversifolia | Dip9 | Diptera |
| Ros3 | Rosales | Potentilla | villosa | Dip10 | Diptera |
| Ros4 | Rosales | Sibbaldia | procumbens | Hym1 | Hymenoptera |
| Sax1 | Saxifragales | Saxifraga | bronchialis | Hym2 | Hymenoptera |
| Sel1 | Sellaginellales | Selaginella | densa | Hym3 | Hymenoptera |
|  |  |  |  | Hym4 | Hymenoptera |
|  |  |  |  | Hym5 | Hymenoptera |
|  |  |  |  | Lep1-7 | Lepidoptera |
|  |  |  |  | Ort1 | Orthoptera |
